# COVID-19 vaccine effectiveness against symptomatic SARS-CoV-2 infection and severe COVID-19 outcomes from Delta AY.4.2: Cohort and test-negative study of 5.4 million individuals in Scotland

**DOI:** 10.7189/jogh.12.05025

**Published:** 2022-07-09

**Authors:** Steven Kerr, Eleftheria Vasileiou, Chris Robertson, Aziz Sheikh

**Affiliations:** 1 Usher Institute, The University of Edinburgh, Edinburgh, Scotland, UK; 2 University of Strathclyde, Glasgow, United Kingdom; Public Health Scotland, Glasgow, Scotland, UK

## Abstract

**Background:**

In July 2021, a new variant of SARS-CoV-2 (severe acute respiratory syndrome coronavirus 2) in the Delta lineage was detected in the United Kingdom (UK), named AY.4.2 or “Delta plus”. By October 2021, the AY.4.2 variant accounted for approximately 10-11% of cases in the UK. AY.4.2 was designated as a variant under investigation by the UK Health and Security Agency on 20 October 2021. This study aimed to investigate vaccine effectiveness (VE) against symptomatic COVID-19 (Coronavirus disease 2019) infection and COVID-19 hospitalisation/death for the AY.4.2 variant.

**Methods:**

We used the Scotland-wide Early Pandemic Evaluation and Enhanced Surveillance (EAVE-II) platform to estimate the VE of the ChAdOx1, BNT162b2, and mRNA-1273 vaccines against symptomatic infection and severe COVID-19 outcomes in adults. The study was conducted from June 8 to October 25, 2021. We used a test-negative design (TND) to estimate VE against reverse transcriptase polymerase chain reaction (RT-PCR) confirmed symptomatic SARS-CoV-2 infection while adjusting for sex, socioeconomic status, number of coexisting conditions, and splines in time and age. We also performed a cohort study using a Cox proportional hazards model to estimate VE against a composite outcome of COVID-19 hospital admission or death, with the same adjustments.

**Results:**

We found an overall VE against symptomatic SARS-CoV-2 infection due to AY.4.2 of 73% (95% confidence interval (CI) = 62-81) for >14 days post-second vaccine dose. Good protection against AY.4.2 symptomatic infection was observed for BNT162b2, ChAdOx1, and mRNA-1273. In unvaccinated individuals, the hazard ratio (HR) for COVID-19 hospital admission or death from AY.4.2 among community detected cases was 1.77 (95% CI = 1.02-3.07) relative to unvaccinated individuals who were infected with Delta, after adjusting for multiple potential confounders. VE against AY.4.2 COVID-19 admissions or deaths was 87% (95% CI = 74-93) >28 days post-second vaccination relative to unvaccinated.

**Conclusions:**

We found that AY.4.2 was associated with an increased risk of COVID-19 hospitalisations or deaths in unvaccinated individuals compared with Delta and that vaccination provided substantial protection against symptomatic SARS-CoV-2 and severe COVID-19 outcomes following Delta AY.4.2 infection. High levels of vaccine uptake and protection offered by existing vaccines, as well as the rapid emergence of the Omicron variant may have contributed to the AY.4.2 variant never progressing to a variant of concern.

The AY.4.2 variant, commonly known as “Delta plus”, is a sub-lineage of the Delta variant of concern (VOC). The first case of AY.4.2 confirmed by viral sequencing in Scotland occurred on June 8, 2021. By the week of October 18 to October 24, 2021, AY.4.2 was present in 11.3% of Delta cases in the UK [1]. As of October 25, 2021, AY.4.2 sequences had been uploaded to the Global initiative on sharing avian influenza data from 42 countries [2]. There was a total of 121 111 confirmed AY.4.2 cases in the UK by January 19, 2022 [3].

The rapid increase in the proportion of cases due to the AY.4.2 variant led the UK Health and Security Agency to designate it as a variant under investigation on October 20, 2021 [1]. There has been significant policy-making interest in the virulence of the AY.4.2 variant, as well as the effectiveness of existing vaccines against it.

We aimed to investigate vaccine effectiveness (VE) against infection and hospitalisation/death due to the AY.4.2 variant. We are not aware of any other studies investigating this topic. We used the Scotland-wide Early Pandemic Evaluation and Enhanced Surveillance (EAVE-II) platform, which consists of linked primary care, secondary care, mortality, virological sequencing, and coronavirus disease 2019 (COVID-19) testing data for the population of Scotland.

We carried out a test-negative design (TND) study of VE against symptomatic infection with AY.4.2. One of the main confounders in studies of this type is that propensity to be tested for COVID-19 may vary by vaccination status. The TND seeks to control for this by matching positive-testing individuals with negative-testing individuals. We also carried out a cohort study of VE against a composite outcome of COVID-19 hospitalisation or death using a Cox proportional hazards model. To isolate hospital admissions due to COVID-19 rather than with COVID-19, we restricted to individuals who tested positive in the community and were not in a hospital at the time of the test, and whose hospital admission was classed as an emergency.

## METHODS

### Study design and population

Early Pandemic Evaluation and Enhanced Surveillance (EAVE II) is a COVID-19 surveillance platform that comprises linked primary care, secondary care, mortality, virological sequencing, and COVID-19 testing data covering 5.4 million (~99% population coverage) people in Scotland. EAVE II has been used to track and forecast the epidemiology of COVID-19, inform deliberations on risk stratification, and investigate vaccine effectiveness and safety [4-13].

We used the EAVE II platform to undertake a TND and cohort analysis of all individuals in Scotland who tested positive for SARS-CoV-2 in the community from June 8 to October 25, 2021. This was done to describe the demographic profile of COVID-19 cases, and to investigate the risk of symptomatic SARS-CoV-2 (severe acute respiratory syndrome coronavirus 2) infection and COVID-19 emergency hospital admission or death.

Individuals entered the TND and cohort studies at the date of specimen collection for a positive test that was virally sequenced and were followed up until the occurrence of the outcome of interest (i.e., symptomatic SARS-CoV-2 infection, COVID-19 emergency hospital admission or death) or the end of the study (October 25, 2021).

### Outcomes

A symptomatic SARS-CoV-2 infection was defined as a positive reverse transcriptase polymerase chain reaction (RT-PCR) test result taken from individuals who presented with COVID-19 symptoms at the time of being tested in the community. We defined a COVID-19 hospital admission as a hospital admission within 14 days of a positive RT-PCR COVID-19 case flagged as an emergency. We defined a COVID-19 death as death within 28 days of a positive RT-PCR test for SARS-CoV-2, or death with COVID-19 recorded on the death certificate. Sequencing data were used to determine the viral variant/sub-variant associated with a COVID-19 infection.

### Statistical analysis

For the test-negative design study, individuals with COVID-19 symptoms and a positive RT-PCR test were defined as cases while those with COVID-19 symptoms but a negative RT-PCR test were defined as controls. A 1:1 ratio was used for cases and controls. The first positive test was selected from individuals with multiple positive tests and a random negative test was selected from individuals with multiple negative tests during the same study period. We prioritised positive over negative test results in study participants with both test results and removed their negative results from the study sample.

We restricted the cohort analysis to individuals who tested positive in the community, whose sample was sequenced, and who were not in a hospital at the time of the test.

Odds ratios (OR) of SARS-CoV-2 infection were estimated via generalised additive logistic regression models. Hazard ratios (HR) for emergency COVID-19 hospital admission or death were estimated using Cox proportional hazard models.

Vaccine status at the time of specimen collection was categorised by a first or second dose of BNT162b2, ChAdOx1, or mRNA-1273 vaccines and time period, demarcated by 14 days elapsed since vaccination for symptomatic infection, and 28 days for COVID-19 hospitalisation or death. For symptomatic SARS-CoV-2 infection, a 14-day time period between vaccination and infection was used because COVID-19 symptoms tend to appear 2-14 days after exposure to the virus [14]. However, a longer time period is typically observed between exposure to SARS-CoV-2 and COVID-19 hospitalisation or death. Therefore, we used a time period of 28 days between vaccination and severe COVID-19 outcomes in this study. Demographic, clinical, and other population characteristics were determined from primary care records, which were extracted in December 2020.

Sex, socioeconomic status, number of comorbid risk groups, age, and calendar time were adjusted for in both analyses. The number of comorbid risk groups was determined from predictor variables in the QCOVID risk prediction algorithm and included as a categorical variable with levels 0, 1, 2, 3, 4, 5+ [15]. QCOVID is a tool for predicting the risk of COVID-19 hospitalisation and death and has been used to inform UK policies on vaccine prioritisation. Socioeconomic status was measured using quintiles of the Scottish Index of Multiple Deprivation (SIMD), where quintile 1 refers to most deprived and quintile 5 to least deprived individuals [16]. Time in days and age were included in the regression models as splines. ORs and HRs together with their 95% confidence intervals (CIs) were derived from the regression coefficients of the logistic regression and Cox models for vaccination status. All analysis was carried out using R version 3.6.1 (Foundation for Statistical Computing, Vienna, Austria).

### Ethics and permission

Data approvals were obtained from the National Research Ethics Service Committee, Southeast Scotland 02 (reference number: 12/SS/0201) and Public Benefit and Privacy Panel for Health and Social Care (reference number: 1920-0279).

### Reporting

This study is reported in accordance with the REporting of studies Conducted using Observational Routinely-collected Data (RECORD) guidelines (S1 Checklist in the **Online Supplementary Document**) [17,18].

## RESULTS

**Figure 1:**
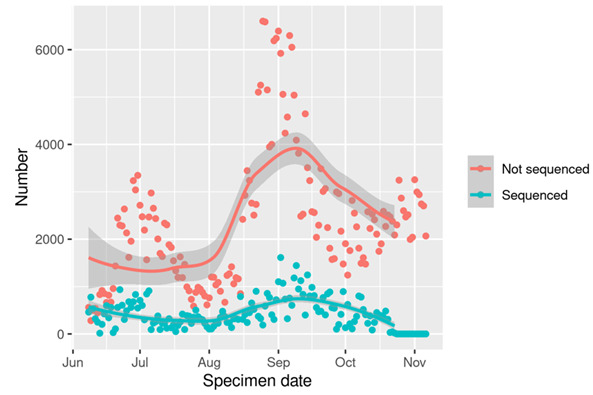
Number of positive tests sequenced and not sequenced per day in Scotland.

**Figure 2:**
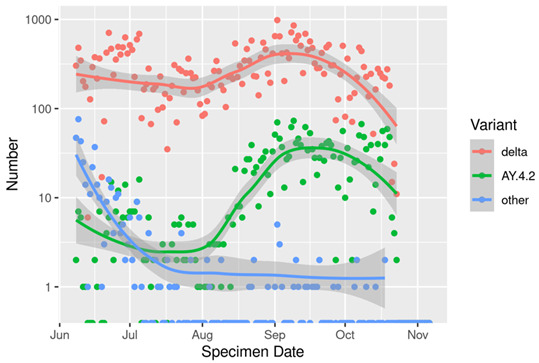
Number of positive tests due to AY.4.2, Delta, and other variants per day in Scotland, among the PCR tests sequenced.

**Figure 3:**
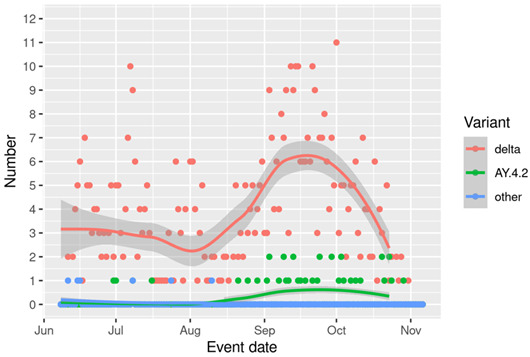
Number of COVID-19 emergency hospital admission or deaths by variant per day in Scotland deriving from individuals with sequenced PCT tests.

**Figure 1** shows the number of positive tests per day by whether the individual was sequenced or not. The number sequenced each day varied with the level of infection; at times of high numbers of infections, the proportion sequenced was lower. **Figure 2** shows the number of positive tests per day by variant, showing the increase in AY4.2 around August and September, followed by a decline along with the whole Delta epidemic. **Figure 3** shows the trend in the number of COVID-19 hospitalisations or deaths by variant among those sequenced.

**Table 1**, along with Table S1 and Table S2 in the **Online Supplementary Document**, gives information on several demographic and clinical characteristics among all those who tested positive, all those who tested positive and were virally sequenced, and all who tested positive and were virally sequenced with AY.4.2. 16% of RT-PCR tests received from the Scottish Government’s Lighthouse laboratory underwent genetic sequencing to determine if positive RT-PCR test results were due to AY.4.2. There were 31 hospital admissions and eight deaths associated with AY.4.2. These tables demonstrate that the positive samples selected for sequencing are representative of all positive samples in the community. The only variable where there is a discrepancy is that a greater proportion of sequenced cases are tested in NHS laboratories (12.5%) compared to all positive cases (5.3%), and a consequent impact on hospitalizations and deaths as hospital admission is much more likely following an NHS laboratory test than a community test.

**Table 1:** Characteristics of all individuals with a positive RT-PCT test, all who were sequenced, and those with AY.4.2

Characteristic	Categories	All positive tests (sequenced and not sequenced)	All positive and sequenced tests	Positive test with AY.4.2
Total number (%)		269 719	44 247 (14.1%)	2397 (7.6%)
Sex	Female	137 554 (51.0%)	22 592 (51.1%)	1212 (50.6%)
	Male	132 165 (49.0%)	21 655 (48.9%)	1185 (49.4%)
Age (years)	18-64	242 802 (90.0%)	38 826 (87.7%)	2106 (87.9%)
	65-79	20 985 (7.8%)	3738 (8.4%)	221 (9.2%)
	80+	5932 (2.2%)	1683 (3.8%)	70 (2.9%)
Age	Mean (SD)	41.4 (16.7)	42.2 (17.8)	43.6 (17.3)
Vaccine status	Unvaccinated	58 973 (21.9%)	10 936 (24.7%)	482 (20.1%)
	One vaccine dose 0-27 days before test	14 602 (5.4%)	2751 (6.2%)	47 (2.0%)
	One vaccine dose >28 days before test	35 141 (13.0%)	5490 (12.4%)	242 (10.1%)
	Two vaccine doses 0-27 days before test	15 235 (5.6%)	2319 (5.2%)	91 (3.8%)
	Two vaccine doses >28 days before test	145 768 (54.0%)	22 751 (51.4%)	1 535 (64.0%)
ChAdOx1 nCoV-19	One vaccine dose 0-27 days before test	902 (0.3%)	238 (0.5%)	7 (0.3%)
	One vaccine dose >28 days before test	8520 (3.2%)	1534 (3.5%)	60 (2.5%)
	Two vaccine doses 0-27 days before test	6521 (2.4%)	1258 (2.8%)	23 (1.0%)
	Two vaccine doses >28 days before test	96 387 (35.7%)	14 886 (33.6%)	1056 (44.1%)
mRNA-1273	One vaccine dose 0-27 days before test	2436 (0.9%)	413 (0.9%)	7 (0.3%)
	One vaccine dose >28 days before test	4603 (1.7%)	606 (1.4%)	41 (1.8%)
	Two vaccine doses 0-27 days before test	1180 (0.4%)	144 (0.3%)	11 (0.5%)
	Two vaccine doses >28 days before test	1195 (0.4%)	115 (0.3%)	10 (0.4%)
BNT162b2	One vaccine dose 0-27 days before test	11 264 (4.2%)	2100 (4.7%)	33 (1.4%)
	One vaccine dose >28 days before test	22 018 (8.2%)	3,350 (7.6%)	141 (5.9%)
	Two vaccine doses 0-27 days before test	7534 (2.8%)	917 (2.1%)	57 (2.4%)
	Two vaccine doses >28 days before test	48 186 (17.9%)	7750 (17.5%)	469 (19.6%)
Hospitalized at the time of being tested	No	263 237 (97.6%)	43 756 (98.9%)	2377 (99.2%)
	Yes	6482 (2.4%)	491 (1.1%)	20 (0.8%)
Type of laboratory	Lighthouse (community)	255 467 (94.7%)	36 691 (87.5%)	2151 (92.7%)
	NHS (hospital)	14 252 (5.3%)	5235 (12.5%)	170 (7.3%)
COVID-19 hospital admission	No	260 411 (96.5%)	41 140 (93.0%)	2264 (94.5%)
	Yes	9308 (3.5%)	3107 (7.0%)	133 (5.5%)
COVID-19 emergency hospital admission	No	261 157 (96.8%)	41 351 (93.5%)	2274 (94.9%)
	Yes	8562 (3.2%)	2896 (6.5%)	123 (5.1%)
COVID-19 death	No	268 170 (99.4%)	43 540 (98.4%)	2372 (99.0%)
	Yes	1549 (0.6%)	707 (1.6%)	25 (1.0%)
Any death	No	267 997 (99.4%)	43 477 (98.3%)	2372 (99.0%)
	Yes	1722 (0.6%)	770 (1.7%)	25 (1.0%)

**Table 2** shows the main results from both the TND study of VE against symptomatic COVID-19 infection and the cohort study of VE against hospitalisation or death for the AY4.2 variant. VE against symptomatic SARS-CoV-2 infection due to AY 4.2 was 73% (95% CI = 62-81) for >14 days post-second vaccine dose. In unvaccinated people, the HR for hospitalisation or death for AY.4.2 was 1.77 (95% CI = 1.02-3.06) relative to those who had the Delta variant. We found a VE of 87.2% (95% CI = 74.7-93.5) for those who had received a second dose of any vaccine >28 days before their positive test. Tables S1 and S2 in the **Online Supplementary Document** show the results of the TND and cohort study with vaccination status broken down by the type of vaccine received. Figure S4 in the **Online Supplementary Document** shows HR for hospitalisation or death by age.

**Table 2:** Vaccine effectiveness in preventing symptomatic SARS-CoV-2 infection and COVID-19 emergency hospital admission or death from AY.4.2 stratified by vaccination status*

Symptomatic SARS-CoV-2 infection				
**Vaccination status**	**Number of tests†**	**Number (%) of positive tests‡**	**Adjusted odds ratio (95% CI)**	**Vaccine effectiveness (95% CI)**
Unvaccinated	7723	44 (0.5)	Reference	Reference
One vaccine dose	35 201	207 (0.6)	0.61 (0.44-0.86)	38.9 (14.2-56.5)
Two vaccine doses 0-13 days before test	9160	48 (0.5)	0.41 (0.27-0.63)	59.2 (37.3-73.4)
Two vaccine doses >14 days before test	158 112	992 (0.6)	0.27 (0.19-0.38)	72.8 (61.8-80.6)
**COVID-19 emergency hospital admission or death**				
Hazard ratio with respect to unvaccinated with delta				
**Vaccination status**	**Number of person-years**	**Number of events**	**Adjusted hazard ratio (95% CI)**	**Vaccine effectiveness (95% CI)**
Unvaccinated	28	14	1.77 (1.02-3.06)	-
Hazard ratio with respect to unvaccinated with AY4.2				
One vaccine dose 0-27 days before test	3	0	-	-
One vaccine dose >28 days before test	16	<5	0.22 (0.05-0.98)	77.7 (1.8-94.9)
Two vaccine doses 0-27 days before test	7	0	-	-
Two vaccine doses >28 days before test	86	23	0.13 (0.06-0.25)	87.2 (74.7-93.5)

CI – confidence interval

*SARS-CoV-2 infection refers to AY.4.2 community tested positive individuals with COVID-19 symptoms at the time of test. COVID-19 emergency hospital admission or death outcomes refer to all AY.4.2 community tested positive individuals. Hazard ratios are not provided for individuals with one or two vaccine doses 0:27 days before test for the delta plus variant due to no COVID-19 emergency hospital admissions or deaths observed in this vaccination category. Full results are in Table S3 in the **Online Supplementary Document**.

†Number of positive and negative tests collected from symptomatic individuals tested in the community.

‡Positive tests refer to all AY.4.2 positive tests collected from symptomatic individuals in the community.

## DISCUSSION

In this study of vaccine effectiveness against symptomatic COVID-19 infection and COVID-19 hospitalisation/death with the AY.4.2 variant, we found that amongst unvaccinated individuals, AY.4.2 was associated with an increased risk of severe COVID-19 outcomes relative to the Delta variant (HR = 1.77, 95% CI = 1.02-3.06). We also found high levels of VE against infection and a composite outcome of COVID-19 hospitalisation or death.

Major strengths of this study include our ability to rapidly access and analyse data on vaccination status, hospitalisation and death records, viral sequencing, and COVID-19 test results from linked databases with national-level coverage of ~5.4 million people. The EAVE-II platform has been used to carry out many research projects related to the epidemiology of COVID-19, as well as vaccine safety and effectiveness. It has been used for forecasting the pandemic [6], risk stratification [7], and vaccine safety [8] and effectiveness [9,10] for emerging variants, including the Delta and Omicron variants [11-13]. In our study of vaccine effectiveness again symptomatic infection, we employed a test-negative design with controls being negative-testing individuals with COVID symptoms to control for propensity to be tested for SARS-CoV-2. In our analysis of serious COVID-19 outcomes, we only counted hospitalisations within 14 days of an RT-PCR positive test that were flagged as an emergency to isolate hospitalisations due to COVID-19, rather than with COVID-19.

However, this study also had some limitations. One concern is that only 16% of positive RT-PCR tests in the study period were sequenced, which raises the possibility of a sample selection bias. To examine this, we looked at the marginal distribution of a number of demographic and clinical variables amongst all who tested positive, all who tested positive and were virally sequenced, and all who tested positive, were virally sequenced, and had AY.4.2. We did not find any large disparities between these groups. There were low numbers of people who had a COVID-19 hospitalisation/death and that were virally sequenced during the study period. This precluded us from estimating VE against serious COVID-19 outcomes in some categories.

## CONCLUSIONS

Although the AY.4.2 variant was designated as a variant under investigation on October 20, 2021, it has never been classified as a variant of concern. Our findings indicate high levels of vaccine effectiveness against symptomatic SARS-CoV-2 infection and severe COVID-19 outcomes associated with the AY.4.2 variant. This, combined with high levels of vaccine uptake across the UK, may have contributed to AY.4.2 never progressing to a variant of concern. At the same time, a new variant called B.1.1.529 or Omicron, was first detected in Scotland by viral sequencing on November 24, 2021, and became the dominant variant within a month. The AY.4.2 variant may have been “outcompeted” by Omicron. This may have also led to relatively little research output on the AY.4.2 variant compared to Omicron. In conclusion, we found that unvaccinated individuals were more susceptible to COVID-19 hospitalisation/death if infected with AY.4.2 compared to the Delta variant, and high levels of VE against both infection and serious COVID-19 outcomes for the AY.4.2 variant.

## Additional material


Online Supplementary Document
