# Supplementary material for: COVID-19 vaccine effectiveness against symptomatic SARS-CoV-2 infection and severe COVID-19 outcomes from Delta AY.4.2: Cohort and test-negative study of 5.4 million individuals in Scotland
Source: J Glob Health. 2022 Jul 9;12:05025. doi: 10.7189/jogh.12.05025 (PMC9269984; doi:10.7189/jogh.12.05025)
Supplement: Online Supplementary Document [file jogh-12-05025-s001.pdf]

Supplementary Information

Table S1: Further characteristics of all individuals with a positive RT-PCT test, all who were sequenced, and those with AY.4.2

Characteristic	Categories	All positive tests (sequenced & not sequenced)	All positive and sequenced tests	Positive test with AY.4.2
Socioeconomic status†	1 (low)	62,783 (23.3%)	10,763 (24.5%)	594 (24.9%)
	2	57,061 (21.2%)	9,551 (21.7%)	512 (21.5%)
	3	48,785 (18.1%)	7,825 (17.8%)	435 (18.2%)
	4	49,085 (18.2%)	7,966 (18.1%)	433 (18.2%)
	5 (high)	50,254 (18.6%)	7,831 (17.8%)	410 (17.2%)
Urban-rural residence	Large Urban Areas	100,301 (37.2%)	16,701 (37.7%)	984 (41.1%)
	Other Urban Areas	106,565 (39.5%)	17,148 (38.8%)	889 (37.1%)
	Accessible Small Towns	23,226 (8.6%)	3,824 (8.6%)	212 (8.8%)
	Remote Small Towns	8,657 (3.2%)	1,453 (3.3%)	74 (3.1%)
	Accessible Rural	21,460 (8.0%)	3,527 (8.0%)	148 (6.2%)
	Remote Rural	7,759 (2.9%)	1,283 (2.9%)	77 (3.2%)
	Unknown	1,751 (0.6%)	311 (0.7%)	13 (0.5%)
Number of coexisting conditions	0	166,657 (61.8%)	26,475 (61.1%)	1,420 (60.5%)
	1	69,533 (25.8%)	11,230 (25.9%)	628 (26.7%)
	2	18,901 (7.0%)	3,380 (7.8%)	191 (8.1%)
	3	5,719 (2.1%)	1,229 (2.8%)	65 (2.8%)
	4	2,243 (0.8%)	548 (1.3%)	26 (1.1%)
	5+	1,430 (0.5%)	458 (1.1%)	19 (0.8%)
Number of RT-PCR tests before specimen date	0	264,448 (98.0%)	42,977 (99.2%)	2,335 (99.4%)
	1	35 (0.0%)	343 (0.8%)	14 (0.6%)
Average household age	Mean (SD)	37.7 (16.2)	38.8 (17.3)	39.5 (17.0)
Size of household	1	61,977 (23.0%)	10,674 (24.6%)	600 (25.5%)
	2	64,597 (23.9%)	10,442 (24.1%)	585 (24.9%)
	3-5	125,625 (46.6%)	20,018 (46.2%)	1,046 (44.5%)
	6-10	11,367 (4.2%)	1,889 (4.4%)	105 (4.5%)
	11-30	598 (0.2%)	163 (0.4%)	9 (0.4%)
	31-100	376 (0.1%)	118 (0.3%)	4 (0.2%)
	101+	178 (0.1%)	46 (0.1%)	0 (0.0%)
Body Mass Index	Underweight	2,616 (1.0%)	452 (1.0%)	20 (0.9%)

	Normal weight	31,616 (11.7%)	5,369 (12.4%)	266 (11.3%)
	Overweight	187,476 (69.5%)	30,210 (69.7%)	1,643 (69.9%)
	Obese	42,775 (15.9%)	7,289 (16.8%)	420 (17.9%)

Table S2: Comorbidities of all individuals with a positive RT-PCT test, all who were sequenced, and those with AY.4.2

Characteristic	Categories	All positive tests (sequenced & not sequenced)	All positive and sequenced tests	Positive test with AY.4.2
Atrial fibrillation	No	260,965 (96.8%)	42,519 (98.2%)	2,314 (98.5%)
	Yes	3,518 (1.3%)	801 (1.8%)	35 (1.5%)
Asthma	No	223,949 (83.0%)	36,642 (84.6%)	1,978 (84.2%)
	Yes	40,534 (15.0%)	6,678 (15.4%)	371 (15.8%)
Blood cancer	No	263,589 (97.7%)	43,124 (99.5%)	2,337 (99.5%)
	Yes	894 (0.3%)	196 (0.5%)	12 (0.5%)
Heart failure	No	262880 (97.5%)	42,929 (99.1%)	2332 (99.3%)
	Yes	1603 (0.6%)	391 (0.9%)	17 (0.7%)
Cerebral palsy	No	264211 (98.0%)	43,274 (99.9%)	2,348 (100.0%)
	Yes	272 (0.1%)	46 (0.1%)	1 (0.0%)
Coronary heart disease	No	257,401 (95.4%)	41,820 (96.5%)	2,273 (96.8%)
	Yes	7,082 (2.6%)	1,500 (3.5%)	76 (3.2%)
Cirrhosis	No	263,348 (97.6%)	43,098 (99.5%)	2,338 (99.5%)
	Yes	1,135 (0.4%)	222 (0.5%)	11 (0.5%)
Congenital heart disease	No	262,445 (97.3%)	43,022 (99.3%)	2,329 (99.1%)
	Yes	2,038 (0.8%)	298 (0.7%)	20 (0.9%)
COPD	No	259,839 (96.3%)	42,287 (97.6%)	2,304 (98.1%)
	Yes	4,644 (1.7%)	1,033 (2.4%)	45 (1.9%)
Dementia	No	263,400 (97.7%)	42,926 (99.1%)	2,328 (99.1%)
	Yes	1,083 (0.4%)	394 (0.9%)	21 (0.9%)
Diabetes 1	No	263,026 (97.5%)	43,092 (99.5%)	2,327 (99.1%)
	Yes	1457 (0.5%)	228 (0.55)	22 (0.9%)
Diabetes 2	No	253,463 (94.0%)	41,177 (95.1%)	2,233 (95.1%)
	Yes	11,020 (4.1%)	2,143 (4.9%)	116 (4.9%)
Epilepsy	No	261,132 (96.8%)	42,716 (98.6%)	2,315 (98.6%)
	Yes	3,351 (1.2%)	604 (1.4%)	34 (1.4%)
Fracture	No	253,607 (94.0%)	41,375 (95.5%)	2,245 (95.6%)
	Yes	10,876 (4.0%)	1,945 (4.5%)	104 (4.4%)
Neurological disorder	No	263,674 (97.8%)	43,173 (99.7%)	2,343 (99.7%)
	Yes	809 (0.3%)	147 (0.3%)	6 (0.3%)
Parkinson’s	No	264,229 (98.0%)	43,255 (99.8%)	2,347 (99.9%)
	Yes	254 (0.1%)	65 (0.2%)	2 (0.1%)

Pulmonary hypertension	No	264,171 (97.9%)	43,227 (99.8%)	2,342 (99.7%)
	Yes	312 (0.1%)	93 (0.2%)	7 (0.3)
Pulmonary rare	No	263,576 (97.7%)	43,136 (99.6%)	2,340 (99.6%)
	Yes	907 (0.3%)	184 (0.4%)	9 (0.4%)
Peripheral vascular disease	No	263,189 (97.6%)	43,033 (99.3%)	2,338 (99.6%)
	Yes	1,294 (0.5%)	287 (0.7%)	11 (0.5%)
Rheumatoid arthritis or SLE	No	262,216 (97.2%)	42,934 (99.1%)	2,327 (99.1%)
	Yes	2,267 (0.8%)	386 (0.9%)	22 (0.9%)
Respiratory cancer	No	264,128 (97.9%)	43,238 (99.8%)	2,349 (100.0%)
	Yes	355 (0.1%)	82 (0.2%)	0 (0.0%)
Severe mental illness	No	234,956 (87.1%)	38,271 (88.3%)	2,071 (88.2%)
	Yes	29,527 (10.9%)	5,049 (11.7%)	278 (11.8%)
Sickle cell disease	No	264,317 (98.0%)	43,287 (99.9%)	2,348 (100.0%)
	Yes	166 (0.1%)	33 (0.1%)	1 (0.0%)
Stroke/TIA	No	260,524 (96.6%)	42,391 (97.9%)	2,308 (98.3%)
	Yes	3,960 (1.5%)	929 (2.1%)	41 (1.7%)
Thrombosis or pulmonary embolus	No	261,362 (96.9%)	42,676 (98.5%)	2,307 (98.2%)
	Yes	3,121 (1.2%)	644 (1.5%)	42 (1.8%)
Care housing category	No	263,763 (97.8%)	43,080 (99.4%)	2,337 (99.5%)
	Yes	512 (0.2%)	202 (0.5%)	10 (0.4%)
	Homeless	208 (0.1%)	38 (0.1%)	2 (0.1%)
Learning disability	No	261,258 (96.9%)	42,722 (98.6%)	2,316 (98.6%)
	Yes	3,173 (1.2%)	587 (1.4%)	31 (1.3%)
Down’s syndrome	No	264,431 (100.0%)	43,309 (100.0%)	2347 (99.9%)
	Yes	53 (0.0%)	11 (0.0%)	2 (0.1%)
Kidney disease	No serious kidney disease	259,529 (96.2%)	42,110 (97.2%)	2,289 (97.4%)
	CKD5 without dialysis or transplant	4,465 (1.7%)	1,060 (2.4%)	51 (2.2%)
	CKD5 with dialysis in the last 12 months	212 (0.1%)	64 (0.1%)	5 (0.2%)
	CKD5 with transplant	277 (0.1%)	86 (0.2%)	4 (0.2%)
Smoking status	Non-smoker	128,685 (47.7%)	20,520 (47.4%)	1,139 (48.5%)
	Ex-smoker	33,140 (12.3%)	5,499 (12.7%)	307 (13.1%)
	Smoker	57,684 (21.4%)	9,778 (22.6%)	516 (22.0%)
	Unknown	44,973 (16.7%)	7,523 (17.4%)	387 (16.5%)
Blood pressure	No investigation	64,044 (23.7%)	10,509 (24.3%)	508 (21.6%)
	Low	4,792 (1.8%)	772 (1.8%)	45 (1.9%)
	Normal	169,003 (62.7%)	27,595 (63.7%)	1,545 (65.8%)
	High	22,353 (8.3%)	3,702 (8.5%)	193 (8.2%)
	Very high	4,291 (1.6%)	742 (1.7%)	58 (2.5%)

† Deprivation status: Quintiles of Scottish Index of Multiple Deprivation

Comorbidities are those used as predictors in the QCOVID algorithm. Ref: Clift AK, et al. Living risk prediction algorithm (QCOVID) for risk of hospital admission and mortality from coronavirus 19 in adults: national derivation and validation cohort study. BMJ 2020;371:m3731.

Table S3: Vaccine effectiveness against symptomatic SARS-CoV-2 infection caused by AY.4.2 stratified by vaccine type

**Vaccine type**	**Vaccine status**	**Number of tests**	**Number of positive tests**	**Odds ratio (95% CI)**	**Vaccine effectiveness (95% CI)**
ChAdOx1 nCoV-19	Unvaccinated	7,723	44	Reference	Reference
	One vaccine dose	6,391	41	0.71 (0.46-1.11)	28.7 (-10.9-54.2)
	Two vaccine doses 0-13 days before test	2,272	<5	0.17 (0.04-0.69)	83.4 (31.1-96.0)
	Two vaccine doses ≥14 days before test	77,905	683	0.46 (0.32-0.64)	54.5 (35.7-67.8)
mRNA-1273	Unvaccinated	7,723	44	Reference	Reference
	One vaccine dose	5,875	38	0.61 (0.39-0.95)	39.5 (4.9-61.5)
	Two vaccine doses 0-13 days before test	1,507	7	0.30 (0.13-0.67)	70.1 (32.8-86.7)
	Two vaccine doses ≥14 days before test	6,436	8	0.06 (0.03-0.14)	93.7 (86.4-97.1)
BNT162b2	Unvaccinated	7,723	44	Reference	Reference
	One vaccine dose	22,935	128	0.61 (0.43-0.88)	38.8 (12.5-57.2)
	Two vaccine doses 0-13 days before test	5,381	37	0.50 (0.31-0.79)	50.2 (21.1-68.6)
	Two vaccine doses ≥14 days before test	73,771	301	0.21 (0.15-0.30)	78.7 (69.9-85.0)

In the cohort analysis, the hazard ratio (HR) for COVID-19 hospitalisation or death was 1.77 (1.02-3.07) for AY4.2 compared to delta in those who were unvaccinated.

Table S4: Hazard ratios and 95% confident intervals (CI) for COVID-19 emergency hospital admission or death from community testing for all categorical variables in the Cox proportional hazards model

**Population characteristics**		**Number of person-years**	**Number of events**	**Hazard Ratios (95% CI)**
Sex				
Female		1,431	296	Reference
Male		1,387	307	1.12 (0.95-1.32)
Socioeconomic status†				
1 (most deprived)		658	167	Reference
2		598	158	1.13 (0.90-1.41)
3		491	102	0.94 (0.73-1.21)
4		521	108	1.02 (0.79-1.31)
5 (least deprived)		532	66	0.64 (0.48-0.86)
Number of coexisting conditions				
No at-risk condition		1,800	226	Reference
One condition		716	174	1.74 (1.43-2.14)
Two conditions		179	89	2.69 (2.07-3.49)
Three conditions		45	55	4.49 (3.22-6.25)
Four conditions		13	20	4.72 (2.89-7.70)
Five or more conditions		7	26	8.48 (5.35-13.42)
Vaccination status by variant				
Hazard ratios with respect to unvaccinated with delta				
Unvaccinated	AY.4.2	28	14	1.77 (1.02-3.07)
	Other	16	<5	1.27 (0.46-3.49)
Hazard ratios with respect to unvaccinated with indicated variant				
	Delta	190	18	0.42 (0.25-0.69)

One vaccine dose 0-27 days before test	AY.4.2	3	0	-
	Other	5	<1	0.56 (0.06-5.01)
One vaccine dose ≥28 days before test	Delta	354	44	0.35 (0.25-0.49)
	AY.4.2	16	<5	0.22 (0.05-0.98)
	Other	3	0	-
Two vaccine doses 0-27 days before test	Delta	155	14	0.20 (0.11-0.35)
	AY.4.2	7	0	-
	Other	2	0	-
Two vaccine doses ≥28 days before test	Delta	1,176	300	0.21 (0.16-0.26)
	AY.4.2	86	23	0.13 (0.06-0.25)
	Other	3	<5	0.19 (0.02-1.73)
† Deprivation status: Quintiles of Scottish Index of Multiple Deprivation				

Figure S1: Hazard ratios for COVID-19 emergency hospital admission or death from community testing by age

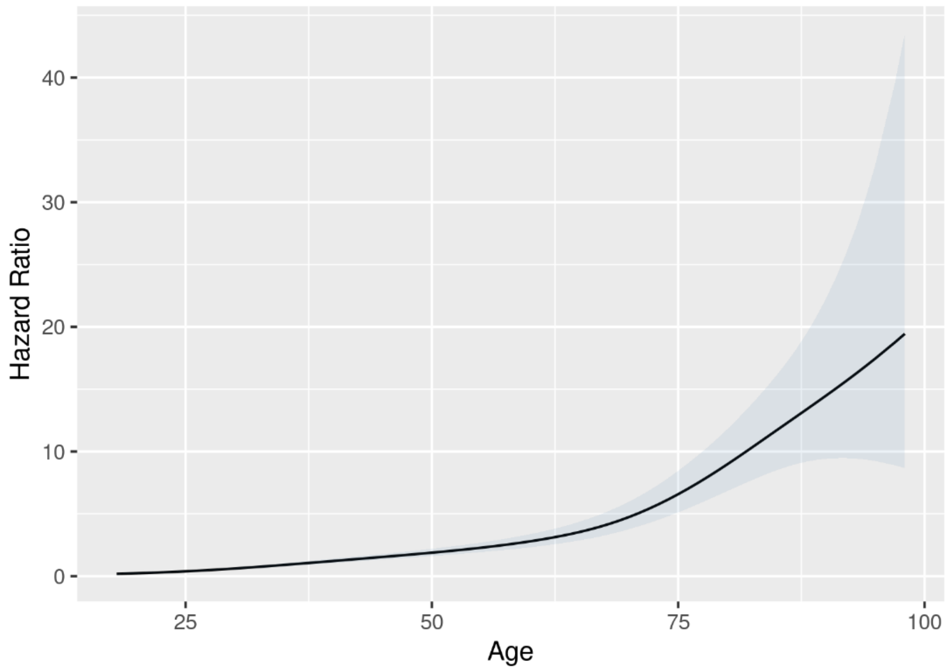

Table S5: STROBE and RECORD statements

	Item No.	STROBE items	Location in manuscript where items are reported	RECORD items	Location in manuscript where items are reported
**Title and abstract**					
	1	(a) Indicate the study's design with a commonly used term in the title or the abstract (b) Provide in the abstract an informative and balanced summary of what was done and what was found	(a) Title:"COVID-19 vaccine effectiveness against symptomatic SARS-CoV-2 infection and severe COVID-19 outcomes from Delta AY.4.2: Cohort and test-negative study of 5.4 million individuals in Scotland "<br><br>(b) Abstract, page 2	RECORD 1.1: The type of data used should be specified in the title or abstract. When possible, the name of the databases used should be included.<br><br>RECORD 1.2: If applicable, the geographic region and timeframe within which the study took place should be reported in the title or abstract.<br><br>RECORD 1.3: If linkage between databases was conducted for the study, this should be clearly stated in the title or abstract.	Title:"COVID-19 vaccine effectiveness against symptomatic SARS-CoV-2 infection and severe COVID-19 outcomes from Delta AY.4.2: Cohort and test-negative study of 5.4 million individuals in Scotland "
**Introduction**					
Background rationale	2	Explain the scientific background and rationale for the investigation being reported			Page 3, Introduction
Objectives	3	State specific objectives, including any			Page 3, Introduction

		prespecified hypotheses			
**Methods**					
Study Design	4	Present key elements of study design early in the paper			Page 3, Methods "We used the EAVE II platform to undertake a TND and cohort analysis of all individuals in Scotland who tested positive for SARS-CoV-2 in the community from 8 June – 25 October 2021, to describe the demographic profile of COVID-19 cases, and to investigate the risk of symptomatic SARS-CoV-2 infection and COVID-19 emergency hospital admission or death.
Setting	5	Describe the setting, locations, and relevant dates, including periods of recruitment, exposure, follow-up, and data collection			Page 3, Methods "We used the EAVE II platform to undertake a TND and cohort analysis of all individuals in Scotland who tested positive for SARS-CoV-2 in the community from 8 June – 25 October 2021, to describe the demographic profile of COVID-

					19 cases, and to investigate the risk of symptomatic SARS-CoV-2 infection and COVID-19 emergency hospital admission or death.
Participants	6	*(a) Cohort study* - Give the eligibility criteria, and the sources and methods of selection of participants. Describe methods of follow-up<br>*Case-control study* - Give the eligibility criteria, and the sources and methods of case ascertainment and control selection. Give the rationale for the choice of cases and controls<br>*Cross-sectional study* - Give the eligibility criteria, and the sources and methods of selection of participants<br><br>*(b) Cohort study* - For matched studies, give matching criteria and number of exposed and unexposed<br>*Case-control study* - For matched studies, give matching		RECORD 6.1: The methods of study population selection (such as codes or algorithms used to identify subjects) should be listed in detail. If this is not possible, an explanation should be provided.<br><br>RECORD 6.2: Any validation studies of the codes or algorithms used to select the population should be referenced. If validation was conducted for this study and not published elsewhere, detailed methods and results should be provided.<br><br>RECORD 6.3: If the study involved linkage of databases, consider use of a flow diagram or other graphical display to demonstrate the data linkage process, including the number of individuals with linked data at each stage.	Page 4, Methods: "Individuals entered the TND and cohort studies at the date of specimen collection for a positive test that was virally sequenced, and were followed up until the occurrence of the outcome of interest (i.e., symptomatic SARS-CoV-2 infection, COVID-19 emergency hospital admission or death) or the end of the study (25 October 2021)."

		criteria and the number of controls per case			
Variables	7	Clearly define all outcomes, exposures, predictors, potential confounders, and effect modifiers. Give diagnostic criteria, if applicable.		RECORD 7.1: A complete list of codes and algorithms used to classify exposures, outcomes, confounders, and effect modifiers should be provided. If these cannot be reported, an explanation should be provided.	Page 4-5. Outcomes and Statistical analysis sections.
Data sources/ measurement	8	For each variable of interest, give sources of data and details of methods of assessment (measurement). Describe comparability of assessment methods if there is more than one group			Page 3, Methods: "Early Pandemic Evaluation and Enhanced Surveillance (EAVE II) is a COVID-19 surveillance platform that comprises of linked primary care, secondary care, mortality, virological-sequencing and COVID-19 testing data covering 5.4 million (~99% population coverage) people in Scotland. EAVE II has been used to track and forecast the epidemiology of COVID-19, inform deliberations on risk stratification, and investigate vaccine effectiveness and safety.[4-13]"

Bias	9	Describe any efforts to address potential sources of bias			Page 4-5, Statistical analysis
Study size	10	Explain how the study size was arrived at			Population-wide study – NA.
Quantitati ve variables	11	Explain how quantitative variables were handled in the analyses. If applicable, describe which groupings were chosen, and why			Page 4-5, Statistical analysis
Statistical methods	12	(a) Describe all statistical methods, including those used to control for confounding<br>(b) Describe any methods used to examine subgroups and interactions<br>(c) Explain how missing data were addressed<br>(d) *Cohort study* - If applicable, explain how loss to follow-up was addressed<br>*Case-control study* - If applicable, explain how matching of cases and controls was addressed<br>*Cross-sectional study* - If applicable, describe analytical methods taking account of sampling strategy			Page 4-5, Statistical analysis

		(e) Describe any sensitivity analyses			
Data access and cleaning methods		..		RECORD 12.1: Authors should describe the extent to which the investigators had access to the database population used to create the study population.<br><br>RECORD 12.2: Authors should provide information on the data cleaning methods used in the study.	
Linkage		..		RECORD 12.3: State whether the study included person-level, institutional-level, or other data linkage across two or more databases. The methods of linkage and methods of linkage quality evaluation should be provided.	Page 3, Methods: "Early Pandemic Evaluation and Enhanced Surveillance (EAVE II) is a COVID-19 surveillance platform that comprises of linked primary care, secondary care, mortality, virological-sequencing and COVID-19 testing data covering 5.4 million (~99% population coverage) people in Scotland. EAVE II has been used to track and forecast the epidemiology of COVID-19, inform deliberations on risk stratification, and investigate vaccine

					effectiveness and safety.[4-13]”
**Results**					
Participants	13	(a) Report the numbers of individuals at each stage of the study (*e.g.*, numbers potentially eligible, examined for eligibility, confirmed eligible, included in the study, completing follow-up, and analysed)<br>(b) Give reasons for non-participation at each stage.<br>(c) Consider use of a flow diagram		RECORD 13.1: Describe in detail the selection of the persons included in the study (*i.e.,* study population selection) including filtering based on data quality, data availability and linkage. The selection of included persons can be described in the text and/or by means of the study flow diagram.	Table 1, Tables S1, Table S2
Descriptive data	14	(a) Give characteristics of study participants (*e.g.*, demographic, clinical, social) and information on exposures and potential confounders<br>(b) Indicate the number of participants with missing data for each variable of interest<br>(c) *Cohort study* - summarise follow-up time (*e.g.*, average and total amount)			Table 1, Tables S1, Table S2
Outcome data	15	*Cohort study* - Report numbers of outcome events or summary			Table 2

		measures over time<br>*Case-control study* - Report numbers in each exposure category, or summary measures of exposure<br>*Cross-sectional study* - Report numbers of outcome events or summary measures			
Main results	16	(a) Give unadjusted estimates and, if applicable, confounder-adjusted estimates and their precision (e.g., 95% confidence interval). Make clear which confounders were adjusted for and why they were included<br>(b) Report category boundaries when continuous variables were categorized<br>(c) If relevant, consider translating estimates of relative risk into absolute risk for a meaningful time period			Table 2
Other analyses	17	Report other analyses done—e.g., analyses of subgroups and interactions, and			

		sensitivity analyses			
**Discussion**					
Key results	18	Summarise key results with reference to study objectives			Page 11 Discussion: "In this study of vaccine effectiveness against symptomatic COVID-19 infection and COVID-19 hospitalisation/death with the AY.4.2 variant we found that amongst unvaccinated individuals, AY.4.2 was associated with an increased risk of severe COVID-19 outcomes relative to the Delta variant HR 1.77 (95% CI 1.02-3.06). We also found high levels of VE against infection, and a composite outcome of COVID hospitalisation or death”
Limitations	19	Discuss limitations of the study, taking into account sources of potential bias or imprecision. Discuss both direction and magnitude of any potential bias		RECORD 19.1: Discuss the implications of using data that were not created or collected to answer the specific research question(s). Include discussion of misclassification bias, unmeasured confounding, missing data, and changing eligibility over time, as they pertain to the study being reported.	Page 12 Discussion: “However, this study also had some limitations. One concern is that only 16% of positive RT-PCR tests in the study period were sequenced, which raises the possibility of a sample selection bias. In order to examine this, we looked at the marginal distribution of a

					number of demographic and clinical variables amongst all who tested positive, all who tested positive and were virally sequenced, and all who tested positive, were virally sequenced, and had AY.4.2. We did not find any large disparities between these groups. There were low numbers of people who had a COVID-19 hospitalisation/death and that were virally sequenced during the study period. This precluded us from estimating VE against serious COVID-19 outcomes in some categories.”
Interpretation	20	Give a cautious overall interpretation of results considering objectives, limitations, multiplicity of analyses, results from similar studies, and other relevant evidence			Page 12 Discussion: “In conclusion, we found that unvaccinated individuals were more susceptible to COVID-19 hospitalisation/death if infected with AY.4.2 compared to the Delta variant, and high levels of VE against both infection and serious COVID-19 outcomes for the AY.4.2 variant. “

Generalisability	21	Discuss the generalisability (external validity) of the study results			Page 11 Discussion:
**Other Information**					
Funding	22	Give the source of funding and the role of the funders for the present study and, if applicable, for the original study on which the present article is based			Page 2, Funding Page 5, Role of the funding source
Accessibility of protocol, raw data, and programming code		..		RECORD 22.1: Authors should provide information on how to access any supplemental information such as the study protocol, raw data, or programming code.	Page 6, Data sharing
